# Association of serum angiopoietin-1, angiopoietin-2 and angiopoietin-2 to angiopoietin-1 ratio with heart failure in patients with acute myocardial infarction

**DOI:** 10.3892/etm.2013.893

**Published:** 2013-01-14

**Authors:** SHAOMIN CHEN, LIJUN GUO, BAOXIA CHEN, LIJIE SUN, MING CUI

**Affiliations:** Department of Cardiology, Peking University Third Hospital; Key Laboratory of Cardiovascular Molecular Biology and Regulatory Peptides, Ministry of Health; Key Laboratory of Molecular Cardiovascular Sciences, Ministry of Education, Beijing 100191, P.R. China

**Keywords:** acute myocardial infarction, angiopoietin, heart failure

## Abstract

The aim of the present study was to investigate the association of the serum angiopoietin (Ang)-1 and Ang-2 levels and the Ang-2 to Ang-1 ratio (Ang-2/Ang-1) with heart failure (HF) in patients with acute myocardial infarction (AMI) during hospitalization. The serum Ang-1 and Ang-2 levels of the AMI patients were measured at admission to hospital. The correlations between serum Ang-1, Ang-2 and Ang-2/Ang-1 with HF were examined. Among 103 patients, 20 developed HF during hospitalization. The serum Ang-2 level and Ang-2/Ang-1 were found to be significantly higher in the patients with HF than in the patients without HF (2,203.1±122.0 vs. 2,102.3±114.4 pg/ml, P=0.001 and 11.4±1.6×10^−2^ vs. 10.6±1.1×10^−2^, P=0.007, respectively). Serum Ang-2 level and Ang-2/Ang-1 were negatively correlated with left ventricular ejection fraction (LVEF; r=−0.352, P<0.001 and r=−0.365, P<0.001, respectively) and positively correlated with the natural logarithm of the level of N-terminal pro-B-type natriuretic peptide (LnNT-proBNP, r=0.367, P<0.001 and r=0.304, P=0.003, respectively) and peak cardiac troponin T (cTnT, r=0.421, P<0.001 and r=0.278, P=0.009, respectively). However, the serum Ang-1 level was not found to correlate significantly with LVEF (r= 0.194, P= 0.05), LnNT-proBNP (r=−0.116, P=0.266) or peak cTnT (r=−0.056, P=0.607). In multivariable logistic regression analysis, Ang-2 (P=0.031), Ang-2/Ang-1 (P=0.018) and NT-proBNP (P=0.001) were revealed to be independently associated with HF. The present study reveals that Ang-2 levels and Ang-2/Ang-1 are independent predictors of HF in AMI patients during hospitalization.

## Introduction

Immediate reperfusion in an infarct-related artery is associated with a favorable outcome in the treatment of acute myocardial infarction (AMI). However, specific patients continue to have poor cardiac function following this procedure, which leads to a poor prognosis (1).

Angiopoietin (Ang)-1, a secreted 70-kDa glycoprotein constitutively expressed by pericytes and vascular smooth muscle cells, is a major agonist for the tyrosine kinase receptor Tie-2 (2). Binding of Ang-1 to Tie-2 promotes vessel integrity, inhibits vascular leakage and suppresses inflammatory gene expression (3). Ang-2 is also a secreted 70-kDa glycoprotein and is exclusively expressed by endothelial cells and acts as an antagonist for Tie-2 (4). Ang-2 has been reported to completely disrupt protective Tie-2 signaling in numerous studies (3). Since Ang-1 and Ang-2 are antagonistic ligands, the Ang-2/Ang-1 ratio (Ang-2/Ang-1) reflects the imbalance between them.

Ang-1 and Ang-2 have been revealed to participate in a number of cardiovascular diseases (5–13). Atherosclerotic plaque microvessel density is associated with plaque hemorrhage and rupture (14). In plaques with high microvessel density, the balance between Ang-1 and Ang-2 is in favor of Ang-2, indicating a role for Ang-2 in the development of unstable plaques (5). Clinical studies have reported that higher plasma Ang-2 levels are predictive of myocardial infarction (6,7) and stroke recurrence (8) and are independent of traditional risk factors. Our previous study (9) demonstrated that serum Ang-1, Ang-2 and Ang-2/Ang-1 are elevated in AMI patients. The extent of myocardial damage may correlate with serum Ang-2 and Ang-2/Ang-1, indicating that Ang-2 and Ang-2/Ang-1 are potential biomarkers of the severity of the disease. However, little is known regarding the role of Ang-1, Ang-2 and Ang-2/Ang-1 in predicting outcomes in AMI patients.

Previous studies revealed that Ang-1 binds integrins on cardiomyocytes and plays a cardioprotective role in cardiac remodeling (15,16). In addition, circulating Ang-2 levels have been identified to be elevated in heart failure (HF) patients (17,18), indicating that Ang-2 may participate in the progression of HF. However, whether circulating Ang-1, Ang-2 and Ang-2/Ang-1 are predictive of HF in AMI patients remains unknown. Therefore, the aim of this study was to investigate the association of serum Ang-1, Ang-2 and Ang-2/Ang-1 with HF in AMI patients during hospitalization.

## Materials and methods

### Patient selection

This study included 103 consecutive patients (88 males and 15 females). The average age was 57.6±12.1 years old. Patients were admitted to the Department of Cardiology, Peking University Third Hospital with first ST-segment elevation myocardial infarction (STEMI) between October 2010 and January 2012. STEMI was diagnosed according to the 2004 guidelines of the American College of Cardiology/American Heart Association. All patients received primary percutaneous coronary intervention (PCI) within 12 h from symptom onset. The exclusion criteria were: age >80 years, previous history of myocardial infarction, significant valvular heart disease, peripheral vascular disease, chronic HF, chronic inflammatory diseases, significant kidney or hepatic diseases or tumor. Heart failure was defined as Killip Class ≥II during hospitalization. This study was conducted in accordance with the Declaration of Helsinki and with approval from the Ethics Committee of Peking University Health Science Center. Written informed consent was obtained from all participants.

### Laboratory assays

Venous blood samples were obtained from all subjects at admission to hospital. All samples were collected in vacuum blood collection tubes with clot activator and were immediately placed in 4°C refrigerators. Within 30 min following collection, samples were centrifuged at 3,000 rpm for 10 min at 4°C, divided into aliquots and stored at −80°C until analysis. Repeated freeze-thaw cycles were avoided.

Serum Ang-1 and Ang-2 levels were measured by enzyme-linked immunosorbent assay (ELISA) according to the manufacturer’s instructions (R&D Systems, Minneapolis, MN, USA). The minimal detection limit was 156 pg/ml. These assays were performed by an investigator blinded to the sample sources. The Ang-2/Ang-1 ratio was calculated.

A previous study revealed that N-terminal pro-B-type natriuretic peptide (NT-proBNP) levels in STEMI patients increased markedly within 24 h following successful primary PCI and then decreased (19). Therefore, NT-proBNP was measured 24 h following PCI, using an E170 Elecsys Modular Analytics assay (Roche Diagnostics GmbH, Mannheim, Germany).

### Echocardiography

Each patient underwent echocardiography 24 h following primary PCI, using a GE-Vingmed V echocardiographic machine (Vivid 7; GE Healthcare, Wauwatosa, WI, USA) with a 3.3-MHz multiphase array probe. Left ventricular ejection fraction (LVEF) was obtained using a modified biplane version of Simpson’s method with apical two- and four-chamber views. These examinations were performed by experienced cardiologists.

### Statistical analysis

Comparisons between groups were conducted by Student’s unpaired t-test, Mann-Whitney U test or Chi-square test. For correlation analysis, the NT-proBNP level was transformed to the natural logarithm (Ln). The Spearman or Pearson correlation was used to identify the bivariate correlations. Multivariable logistic regression analyses were performed to determine whether Ang-1, Ang-2 and Ang-2/Ang-1 were independently associated with HF during hospitalization. P<0.05 was considered to indicate a statistically significant difference. All analyses were performed using SPSS for Windows v15.0 (SPSS, Inc., Chicago, IL, USA).

## Results

### Clinical characteristics and laboratory observations

Among the 103 patients included in this study, 20 developed HF during hospitalization (Killip class II, n=16; III, n=3; and IV, n=1).

Table I summarizes the clinical characteristics and laboratory observations of patients with or without HF. Serum Ang-2 levels and Ang-2/Ang-1 ratio were observed to be significantly higher in patients with HF than in patients without HF (2,203.1±122.0 vs. 2,102.3±114.4 pg/ml, P=0.001 and 11.4±1.6×10^−2^ vs. 10.6±1.1×10^−2^, P=0.007). Other variables associated with HF in univariate analysis included gender (male; 70.0 vs. 89.2%, P=0.029), age (64.9±11.7 vs. 55.9±11.6 years, P=0.002), LVEF (48.1±7.3 vs. 53.3±7.6%, P=0.007), NT-proBNP [3,369 (1,112–4,778) vs. 829 (375–1,379) pg/ml, P<0.001] and peak cTnT [6.9 (3.3–10) vs. 3.1 (2.0–5.3) ng/ml, P=0.002].

### Association between serum angiopoietins and clinical variables

**Figure 1.**
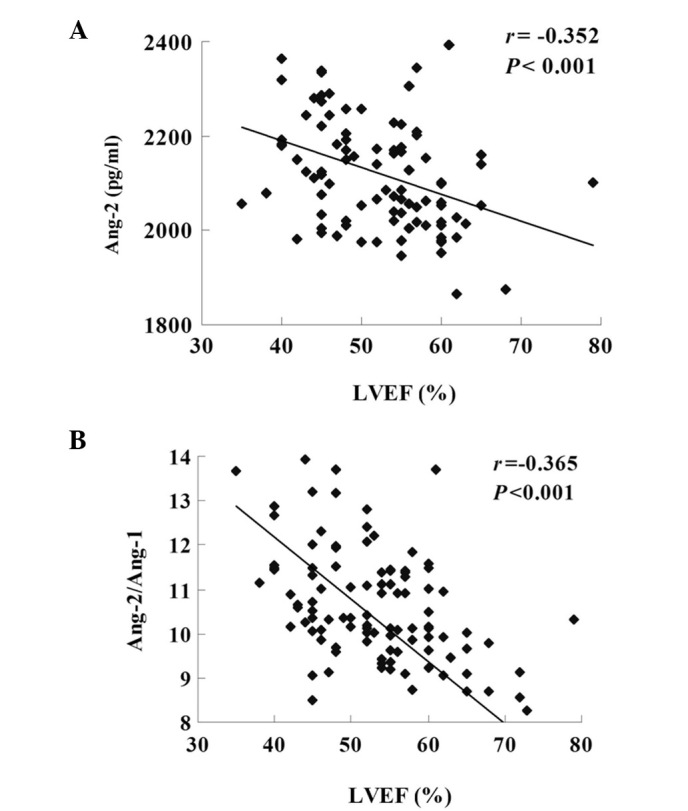
(A) Serum Ang-2 levels and (B) Ang-2/Ang-1 were negatively correlated with LVEF. Ang, angiopoietin; Ang-2/Ang-1, Ang-2 to Ang-1 ratio; LVEF, left ventricular ejection fraction.

**Figure 2.**
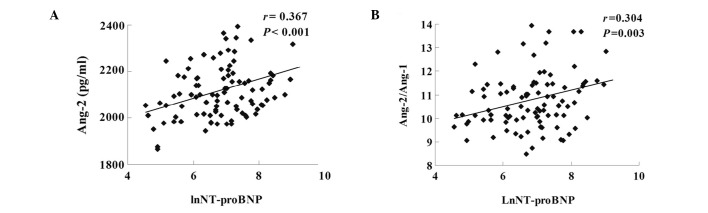
(A) Serum Ang-2 levels and (B) Ang-2/Ang-1 were positively correlated with LnNT-proBNP. Ang, angiopoietin; LnNT-proBNP, logarithmic N-terminal pro-B-type natriuretic peptide.

**Figure 3.**
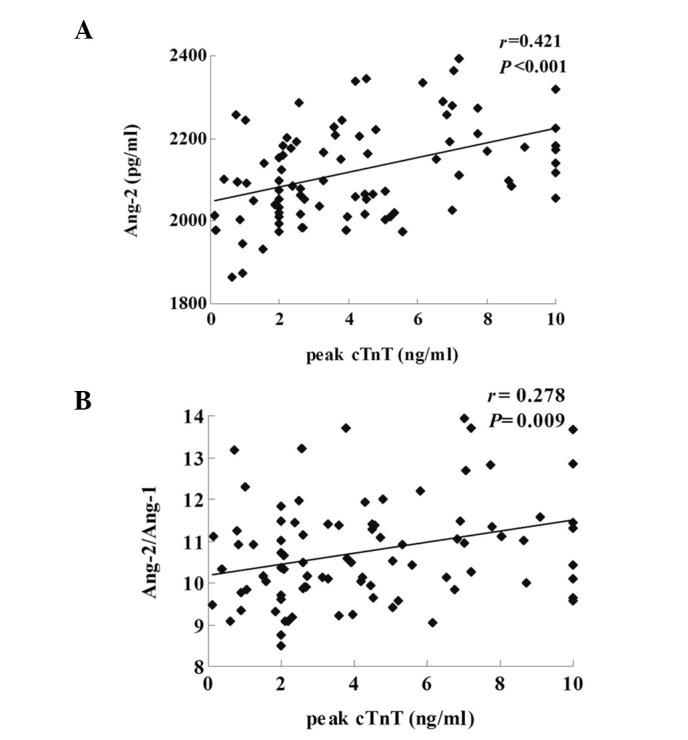
(A) Serum Ang-2 levels and (B) Ang-2/Ang-1 were positively correlated with peak cTnT. Ang, angiopoietin; cTnT, cardiac troponin T.

Serum Ang-2 levels and Ang-2/Ang-1 ratio were negatively correlated with LVEF (r=−0.352, P<0.001 and r=−0.365, P<0.001, respectively; Fig. 1) and positively correlated with LnNT-proBNP (r=0.367, P<0.001 and r=0.304, P=0.003, respectively; Fig. 2) and peak cTnT (r=0.421, P<0.001 and r=0.278, P=0.009, respectively; Fig. 3). However, serum Ang-1 levels were not significantly correlated with LVEF (r=0.194, P=0.05), LnNT-proBNP (r=−0.116, P=0.266) or peak cTnT (r=−0.056, P=0.607). Serum Ang-1, Ang-2 and Ang-2/Ang-1 were not observed to be significantly associated with age, gender, comorbidities (hypertension, diabetes mellitus or hyperlipidemia), smoking status, time from symptom onset to reperfusion, hemodynamic variables (systolic blood pressure or heart rate) or laboratory observations other than LnNT-proBNP and peak cTnT (data not shown).

### Association between serum angiopoietins and heart failure

In the multivariable logistic regression analysis, Ang-2 (P=0.031), Ang-2/Ang-1 (P=0.018) and NT-proBNP (P=0.001) were independently associated with HF, following adjustment for age, gender, hypertension, diabetes mellitus, time from symptom onset to reperfusion, LVEF, serum creatinine, hemoglobin (Hb) level and peak cTnT (Table II).

## Discussion

Ang-1 and Ang-2 are well known to be associated with several forms of cardiovascular (6–14) and inflammatory diseases (20,21). Previous studies revealed that circulating Ang-2 levels and the Ang-2/Ang-1 ratio may be suitable biomarkers of inflammatory diseases. In patients with severe sepsis, circulating Ang-2 levels correlate with markers of endothelial cell activation and 28-day mortality (20). In acute lung injury patients, Ang-2/Ang-1 is an independent predictor of mortality (21).

AMI is also characterized by inflammatory processes and endothelial dysfunction (22). The prognosis for AMI and the preservation of cardiac function have been significantly improved by PCI and proper cardiac care unit management. However, specific patients still exhibit poor cardiac function and clinical course (1). Our previous study (9) demonstrated that serum Ang-1, Ang-2 and Ang-2/Ang-1 are elevated in AMI patients. However, whether circulating Ang-1, Ang-2 and Ang-2/Ang-1 are predictive of HF in AMI patients remains unknown. The current study is the first to investigate the predictive value of Ang1, Ang-2 and Ang2/Ang-1 in AMI patients.

This study identified that the serum Ang-2 levels and the Ang-2/Ang-1 ratio were positively correlated with LnNT-proBNP and negatively correlated with LVEF, indicating that higher Ang-2 and Ang-2/Ang-1 were associated with poor cardiac function in AMI patients. In the multivariable logistic regression analysis, Ang-2 and Ang-2/Ang-1 were independently associated with HF in AMI patients during hospitalization.

First, the extent of myocardial damage may be linked to the level of circulating Ang-2 and the Ang-2/Ang-1 ratio. Myocardial ischemia and reperfusion may not only injure cardiomyocytes, but also cause endothelial activation and injury, which increase vascular permeability and the recruitment of inflammatory cells (22). Ang-1 and Ang-2 play divergent roles in vascular quiescence and inflammation. Ang-2 mediates vascular leakage (23) and inflammation (24), while Ang-1 has anti-permeability (23), anti-inflammatory (25) and cardioprotective effects (15). In an animal myocardial infarction model, adenoviral vectors carrying Ang-2 have been demonstrated to increase infarct size (26), while the administration of adenovirus expressing Ang-1 in an animal ischemia/reperfusion model prevented vascular leakage and cardiomyocyte death and enhanced cardiac function (27).

Second, Ang-1 and Ang-2 may participate in the progression of HF. A previous study by Chong *et al* which included 39 patients with acute HF, 40 patients with chronic HF and 17 healthy controls, identified elevated plasma Ang-2 levels in all HF patients and a significant correlation between Ang-2 and LVEF (17). Eleuteri *et al* revealed that Ang-2 levels are inversely correlated with the 6-min walking test (r=−0.65, P<0.0001) and peak oxygen consumption (r=−0.57, P=0.0002) in patients with chronic HF, indicating that Ang-2 levels progressively increase with hemodynamic and functional decline in these patients (18). In animal cardiac hypertrophy models, Ang-1 reduced the left ventricle/tibia ratio and fibrosis, indicating a cardioprotective role in cardiac remodeling (16).

The present study did not identify a significant correlation between the level of Ang-1 and cardiac function in AMI patients. Consistent with the findings of Chong *et al*(17), plasma Ang-1 levels were not observed to differ significantly between the patients with HF and the healthy controls. We hypothesized that the protective effect of Ang-1 may be overcome by Ang-2.

The sample size of this study is relatively small. Therefore, future studies are required to confirm our findings. Ang-1 binds to integrins on cardiomyocytes and plays a cardioprotective role in cardiac remodeling (16). However, the effect of Ang-2 on cardiac remodeling remains unknown. Further investigation is also required to determine whether Ang-1, Ang-2 and Ang-2/Ang-1 are associated with HF in AMI patients in the chronic phase.

In the present study, serum Ang-1 and Ang-2 were observed to be associated with development of HF in AMI patients in the acute phase. Serum Ang-2 levels and the Ang-2/Ang-1 ratio were identified to be independent predictors of HF during hospitalization.

## Figures and Tables

**Table I.** Clinical characteristics and laboratory observations.

	All patients (n=103)	HF (n=20)	No HF (n=83)	P-value
Male, n (%)	88 (85.4)	14 (70.0)	74 (89.2)	0.029
Age, years	57.6±12.1	64.9±11.7	55.9±11.6	0.002
Hypertension, n (%)	49 (47.6)	12 (60.0)	37 (44.6)	0.319
Diabetes mellitus, n (%)	24 (23.3)	5 (25.0)	19 (22.9)	0.842
Hyperlipidemia, n (%)	46 (44.7)	8 (40.0)	38 (45.8)	0.803
Current smokers, n (%)	80 (77.7)	13 (65.0)	67 (80.7)	0.103
Systolic blood pressure, mmHg	137.4±29.9	136.0±38.7	137.8±27.6	0.819
Heart rate, beats/minute	74.4±14.9	74.4±17.4	74.4±14.3	1.000
Time from symptom onset to reperfusion, min	240 (193–367)	285 (220–400)	230 (180–365)	0.168
Culprit vessel				
LAD, n (%)	57 (55.3)	12 (60.0)	45 (54.2)	0.803
Other vessels, n (%)	46 (44.7)	8 (40.0)	38 (45.8)	
LVEF, %	52.2±7.8	48.1±7.3	53.3±7.6	0.007
NT-proBNP, pg/ml	1,027 (450–1,941)	3,369 (1,112–4,778)	829 (375–1,379)	<0.001
Peak cTnT, ng/ml	3.8 (2.0–6.8)	6.9 (3.3–10)	3.1 (2.0–5.3)	0.002
Hs-CRP, mg/l	6.3 (2.5–15.0)	7.0 (4.7–31.2)	6.2 (2.1–13.2)	0.088
WBC count, 10^9^/l	10.6±3.5	10.2±3.5	10.7±3.5	0.586
Hb, g/L	147.3±16.5	141.8±21.3	148.7±14.9	0.092
Serum creatinine, umol/l	75.8±13.7	76.6±11.6	75.6±14.2	0.782
Ang-1, pg/ml	19,885.0±1,891.3	19,575.5±2,425.5	19,959.6±1,748.5	0.418
Ang-2, pg/ml	2,121.9±122.1	2,203.1±122.0	2,102.3±114.4	0.001
Ang-2/Ang-1, 10^−2^	10.8±1.2	11.4±1.6	10.6±1.1	0.007

Data are presented as mean ± SD, median (inter-quartile range) or percentages. Ang, angiopoietin; Ang-2/Ang-1, Ang-2 to Ang-1 ratio; Hb, hemoglobin; HF, heart failure; Hs-CRP, high-sensitivity-CRP; LAD, left anterior descending artery; LVEF, left ventricular ejection fraction; NT-proBNP, N-terminal pro-B-type natriuretic peptide; cTnT, cardiac troponin T; WBC, white blood cell.

**Table II.** Multivariable predictors of heart failure.

	OR	95% CI	P-value
NT-proBNP	1.001	1.000–1.002	0.001
Ang-2	1.011	1.003–1.019	0.031
Ang-2/1	4.306	1.287–14.406	0.018

Ang, angiopoietin; Ang-2/Ang-1, angiopoietin-2 to angiopoietin-1 ratio; NT-proBNP, N-terminal pro-B-type natriuretic peptide; OR, odds ratio; CI, confidence interval.
